# Description of Sheep Pox Outbreak in Spain in 2022–2023: Challenges Found and Lessons Learnt in Relation with Control and Eradication of This Disease

**DOI:** 10.3390/v16071164

**Published:** 2024-07-19

**Authors:** Cáceres G. Germán, Romero G. Luis, Bonilla G. Sergio, Guerrero C. Fatima, Fernandez M. Manuel, Capilla G. Jaime, Tejero C. Jesús

**Affiliations:** 1 General Subdirectorate of Animal Health, Hygiene and Traceability, Ministry of Agriculture, Fisheries and Food (MAPA), 28010 Madrid, Spain; ljromero@mapa.es (R.G.L.); sbonilla@mapa.es (B.G.S.); fguerrero@mapa.es (G.C.F.); 2 Consejería de Agricultura, Pesca, Agua y Desarrollo Rural de la Junta de Andalucía, 41071 Sevilla, Spain; manuel.fernandez.morente@juntadeandalucia.es; 3 Consejería de Agricultura, Ganadería y Desarrollo Rural de la Junta de Comunidades de Castilla-La Mancha, 45071 Toledo, Spain; jcapilla@jccm.es (C.G.J.); jtejero@jccm.es (T.C.J.)

**Keywords:** Spain, sheep pox virus, outbreak description, control, eradication

## Abstract

Sheep pox and goat pox are infectious viral diseases that affect ovine and caprine animals and are caused by two viruses of the family *Poxviridae*, genus *Capripoxvirus*. Sheep pox has been traditionally endemic in Africa, the Middle East, and several Southeast Asian countries, but it is considered a transboundary disease capable of affecting previously free countries epidemically. It is a disease of compulsory immediate notification to the World Organization for Animal Health (WOAH) and the European Union (EU). On 19 September 2022, the disease reemerged in Spain, which had been free of it since 1968, causing a total of 30 outbreaks until 17 May 2023, when the last outbreak of the disease was reported. The control and eradication measures implemented were those laid down in EU legislation, based on the total stamping out of positive herds, zoning and restriction of movement, and strengthening of biosecurity and passive surveillance. This manuscript describes the outbreak, as well as assesses the challenges and lessons learned in relation to its management, with the aim of helping in the effective management of future outbreaks of this disease.

## 1. Introduction

Sheep pox (SPP) and goat pox (GTP) are infectious viral diseases that affect ovine and caprine animals and are caused by two species of viruses of the family *Poxviridae*, genus *Capripoxvirus* [1]. SPP has been traditionally endemic in Africa (north of the Equator), the Middle East, Turkey, Iran, Afghanistan, India, Nepal, parts of the Republic of China, and Bangladesh. It is considered a transboundary disease capable of crossing national borders and affecting previously free countries epidemically. In recent years, the disease has made epidemic incursions in Southern Europe, affecting Greece and Bulgaria within the EU [1].

In Spain, free from the disease since 1968 [2], its reintroduction was first detected in September 2022, and during the outbreak, sheep and goat farms in the Autonomous Community of Andalusia (AND) and Castilla-La Mancha (CLM) were affected.

On 15 September 2022, the Official Veterinary Services (OVS) of AND were informed of the detection of the presence of skin lesions compatible with SGP in a mixed holding of sheep and goats for meat production, with a census of 314 sheep and 11 goats in the municipality of Benamaurel in the province of Granada, AND. The OVS immediately visited the suspected holding, detecting a total of 50 sheep with SGP-compatible clinical signs. Samples of blood, sera, and from skin lesions were immediately sent to the National Reference Laboratory (NRL), Central Veterinary Laboratory in Algete, Madrid, for laboratorial diagnosis, including for capripoxvirus. Samples were positive by real-time PCR (rPCR) for capripoxvirus and then identified, by a gel-based PCR, to be sheep pox virus (SPV), confirming the disease in Spain after more than 50 years since the disease was eradicated in 1968.

Immediately after confirmation, the OVS of AND implemented control and eradication measures in line with the EU legislation, particularly the Delegated Regulation (EU) 2020/687 of the Commission and the national contingency plan. These measures included the stamping out of the farm with killing of animals, safe disposal of carcasses and risk products in the closest authorized rendering plant under official channeling procedure, and official cleaning and disinfection. The culling of the animals was performed on the spot, taking EU welfare legislation into account to avoid any unnecessary suffering of the animals. Furthermore, Restricted Zones (RZ) were implemented, including a 3 km Protection Zone (PZ) and a 10 km Surveillance Zone (SZ). The measures implemented in the restricted zone included the following:

– Census of all farms and susceptible animals (species, categories, number of animals/establishment).
– Official visits and clinical inspections of all farms every 15 days to early detect any new outbreak.
– Ban on movements of animal and risk products, including reproductive materials and animal by-products.
– Prohibition of fairs, markets, exhibitions, and other concentrations of susceptible animals.
– Enhanced biosecurity and passive surveillance.
– Ban on outdoor grazing of animals in common pastures within the restricted zone.

Official investigations in farms considered at risk due to epidemiological links with the outbreaks were also performed outside the restriction zone.

As we will describe later, supplementarily to the RZ, Further RZs were applied around the RZ to give the restricted areas higher security in terms of virus containment these areas.

In the case of SGP, the European Union legislation allowed for the movement of lambs from healthy farms located within the restricted area to an officially designated slaughterhouse, ideally within the RZ, but if not possible, in the nearest available one, as well as the movement of milk from these farms to a single processing plant where it would be heat-treated though an effective time/temperature. The OVS were required to designate and authorize slaughterhouses and milk treatment plant to do so, as well as to establish controls on movements to ensure traceability of lambs and milk, and on the cleaning and disinfection of vehicles involved in these movements.

## 2. Description of the Outbreak

After the first detection, and in the frame of the epidemiological investigation carried out by the OVS in the affected area of AND, the movement of the lambs was identified from a lamb fattening holding located 200 m from the first confirmed holding in AND, towards an assembly center in the municipality of Villaescusa de Haro in the province of Cuenca, CLM, and located 363 km (road distance) away from the index case. The OVS of CLM carried out a first visit without finding any animals with clinical signs and took samples that were negative, but the holding remained under official control; however, in a second inspection visit a few days later, they detected 1 animal with compatible clinical signs out of a total census of 890 ovine inspected, which was sampled and confirmed as positive in the NRL. Over the following weeks, new outbreaks were confirmed in both the Autonomous Communities, CLM and AND.

On the 16 September 2022, an isolate was identified by specific PCR as a sheep pox virus strain. On 19 September 2022, sequencing analysis determined that it was a field strain, not a vaccine strain [3]. Samples were immediately sent to the EURL (Sciensano) for whole genome sequencing.

During the epidemiological investigations carried out, the source of the virus was never conclusively identified. However, the most plausible hypothesis points to an accidental introduction from North Africa, where the disease is endemic, possibly through people from these countries working in sheep and goat farms in Spain.

The whole sequencing studies of the viral genome detected in the samples of the first outbreak in Benamaurel, Province of Granada, carried out at the European Reference Laboratory (EURL) in Sciensano, Belgium, identified genomic similarities with strains from Morocco (2017), Egypt (2018), and Türkiye (1970) [4]. But these did not allow us to establish more specific conclusions due to the low number of sequences of this virus deposited in the public databases.

In total, in Spain, 30 outbreaks were confirmed from September 2022 to May 2023, when the last outbreak was confirmed in CLM, which meant the slaughter of a total of 51.778 sheep and 767 goats in the frame of the stamping out policy applied for the control of the disease following EU legal frame (Table 1).

In Table 1, we can see that even in cases thought to have been infected for a long time before the first official visit, particularly in outbreak 1/2022 where there were animals with old lesions, and the infection was thought to have been present for more than 1 month before the official first visit, the number of animals reported dead by the farmer was small (30), and they happened progressively; in no case was there an explosive mortality in this farm.

On the other hand, there was a direct relation between the number of animals clinically affected at the time of the first visit and the types of lesions detected in the clinical inspection, given that older lesions were observed, and more animals were affected where the disease had been present for a longer time. This was important, as older lesions meant higher virus shedding, more probability of scabs falling on the floor thus allowing long-term virus stability in the environment (farm, outdoor pastures, etc.) and in summary, more probability of transmission to other farms. In most cases though, the number of animals clinically affected at the first visit was small, which indicated early detection and a short time of presence of the disease before detection. In two cases, the virus was detected by PCR even before the appearance of the first clinical signs, which indicated that the virus was detectable by PCR in saliva swabs during the incubation period.

**Figure 1:**
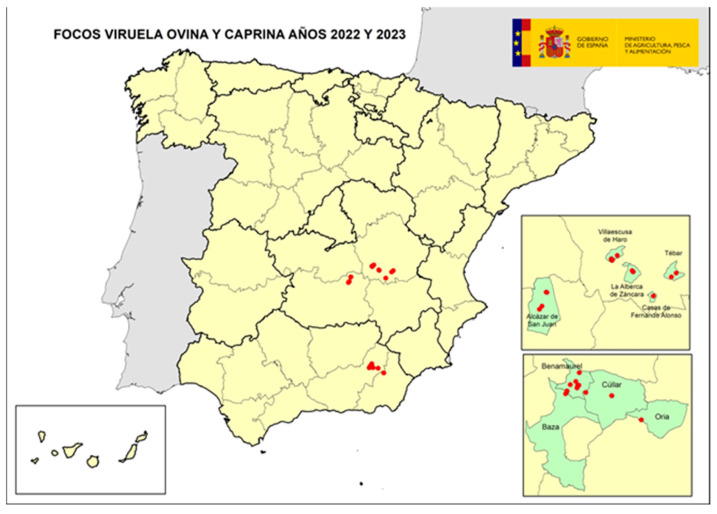
Map with location of total confirmed SGP outbreaks in Spain during the 2022–2023 outbreak (source: OVS AND/CLM, and MAPA).

The following map shows the spatial location of the total 30 outbreaks (Figure 1).

There were important differences between the production systems of sheep and goats in the two affected areas of AND and CLM which produced some differences in the epidemiological evolution of the outbreak.

In the case of AND, the production was characterized by small-/medium-sized farms to produce lambs that were moved to fattening places and then to slaughterhouses. These herds usually went outdoor to graze on the herbaceous resources available; many of these pastures, as well as the paths to reach them, were used by different herds sometimes at the same time, based on the epidemiological investigations carried out in this area, which was the main source of infection for new outbreaks. In total, 13 outbreaks were detected in this Autonomous Community, all located within the previously established Restriction Zones, saving for the outbreak in Oria, Almería, that in any case was located very close to the outer limit of the previous restriction zone. The last outbreak in this area was confirmed in November 2022.

**Figure 2:**
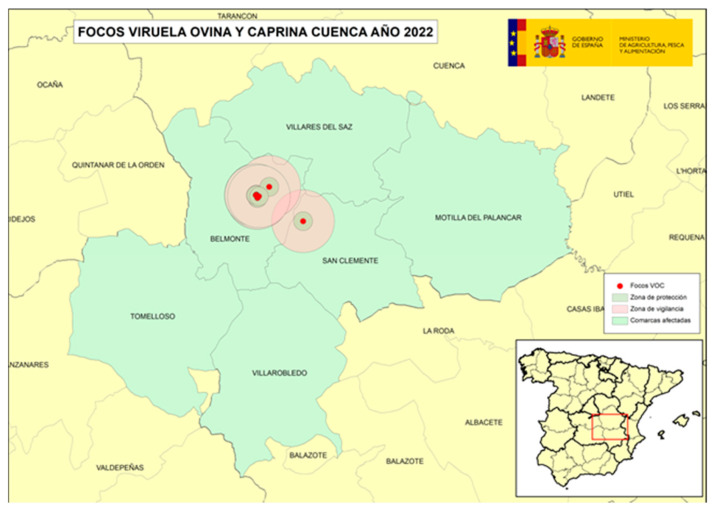
SGP outbreaks in Villaescusa de Haro and Alberca de Záncara, Province of Cuenca, September 2022–November 2022 (source: OVS CLM and MAPA).

**Figure 3:**
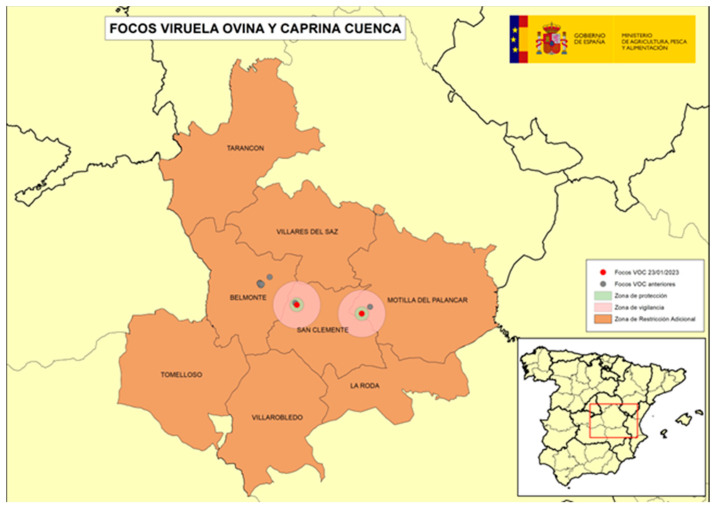
SGP outbreaks in Alberca de Záncara and Tébar, Province of Cuenca, November 2022–January 2023 (source: OVS CLM and MAPA).

**Figure 4:**
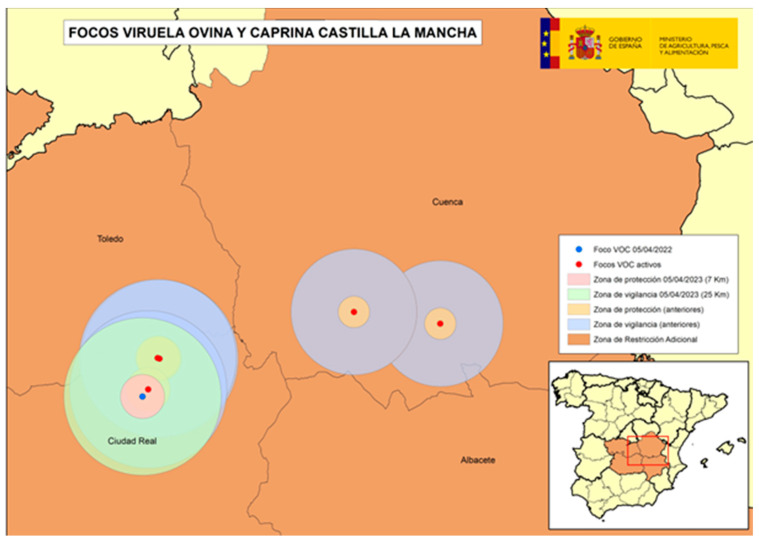
SGP outbreaks in Alcázar de San Juan, Province of Ciudad Real, April 2023 (source: OVS CLM and MAPA).

**Figure 5:**
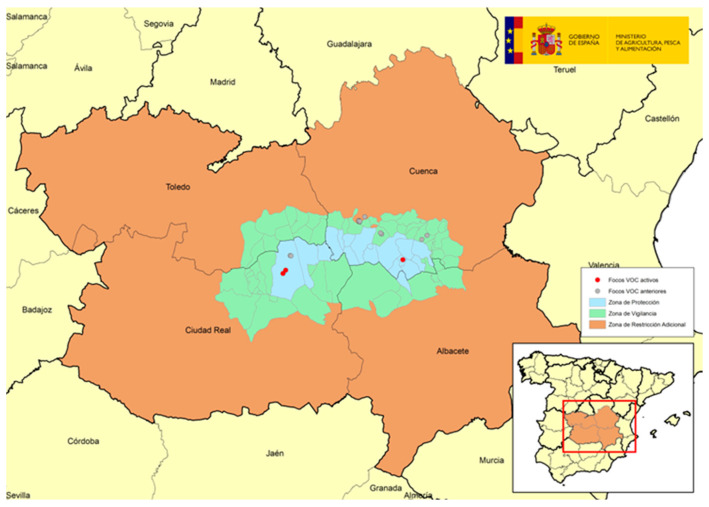
Last SGP outbreak in Casas de Fernando Alonso, Province of Cuenca (May 2023) (source: OVS CLM and MAPA).

On the other hand, in the affected area of CLM, production in general was characterized by large breeding holdings in intensive/semi-extensive regimes aimed at the production of milk, whose lambs went to fattening and assembly centers, and then went to slaughterhouses. Many farms had inventories of thousands of animals and belonged to same operators, who in some cases, managed the entire cycle from breeding farms to assembly centers and fattening places where the lambs were carried to final weight, therefore, few operators handled many lambs in the area. In this area, there was therefore a high number of epidemiological links between many of the farms (same owners, shared staff, common use of machinery, vehicles for collection of lambs, vehicles for milk collection, etc.). In this area, there was a spread pattern where the implication of the human factor was evident, since several long-distance jumps occurred outside the previously established restricted zone, some of them after an abnormally long time since the last previously detected outbreak, which resulted at the end in a total of four successive situations (Figure 2, Figure 3, Figure 4 and Figure 5) which needed the application of restriction zones that increased progressively in space and time during the outbreak, as the previous cluster/restriction zones were maintained under restrictions. Among the factors identified that could have contributed to this spread pattern, and that during epidemiological investigations were thought to be the most plausible source of infection for new outbreaks in this area, were the following: (1) the high number of epidemiological links between holdings, particularly movements of animal transport vehicles collecting lambs between different holdings with access of vehicles into the holdings, possibly in inadequate cleaning and disinfection conditions; (2) delays in reporting compatible clinical signs by some farmers to the OVS of CLM; and (3) low biosecurity level in some of the farms. These factors meant complications in the control and the eradication of the disease in this area that took longer than expected and were desirable. In total, 17 outbreaks were detected in CLM, and the last outbreak in this area was confirmed in May 2023.

It should be noted that these important differences between the production systems of sheep and goats in the two affected areas of AND and CLM determined the differences in the evolution of the outbreak and, likewise, important differences in the challenges found in relation with the disease control, challenges that are analyzed in point 3 of this article.

The following four figures show the spatial evolution of the epidemiological situation and of the restriction zones in Castilla-La Mancha, which were progressively extended in space and time to adapt to the negative evolution of the epidemiological situation, giving rise to four successive restriction zones (MAPA). Figure 2 shows the first outbreaks confirmed in CLM where the RZ was applied in the following manner: a 3 km protection zone for 21 days and a 10 km surveillance zone for 30 days. Figure 3 represents the first jump of the disease outside of the previous restricted zone and the reason for the first enlargement in time and space of the RZ. Figure 4 shows the second jump outside the previous RZ applied, which meant the second enlargement in space and time of the RZ and the application of a surrounding Further RZ and, finally, Figure 5 represents the last outbreak confirmed in CLM and Spain, and the final RZ, composed of a protection zone of 10 km radius for 48 days, and surveillance zone of 30 km radius for 66 days.

## 3. Challenges and Lessons Learned in the Management of the SGP Outbreak 2022–23 in Spain

During the outbreak, important findings and challenges were found by the OVS that could be impacting the effective control of the disease. These challenges were overcome through the implementation of measures which were the key for the effective control of the outbreak, and therefore can be taken as lessons learned for future outbreaks management.

Although there were others, the most relevant challenges were the following:

- Unexpected clinical presentation.
- Sampling strategy in restricted areas and samples of choice for confirmation of the disease.
- Awareness campaigns among farmers and veterinarians are critical for early detection of new outbreaks.
- Farm and animal transport biosecurity is fundamental for the control of the outbreak.
- Strict control on movement of animals in problematic affected areas played a critical role in controlling the outbreak.
- Progressively increasing the size and duration of restricted zones to adapt to the worsening situation was very effective in the control of the outbreak in problematic affected areas.

### 3.1. Unexpected Clinical Presentation

The existing literature describes high mortality at herd level, which could be up to 100% of the animals, more likely in animals without prior immunity [5]. It should be kept in mind that this information was possibly based on the experience of affected countries that were mainly in Africa, north of the equator, the Middle East and Asia, countries where the emergency killing of the total affected herds is normally not used as a control measure, so the disease evolves in the herd, and therefore the aggregated mortality is very high after a period of time when the disease is present in the herd.

Contrary to the description above, in Spain, the experience has been different regarding the clinical presentation of the disease, both at herd and animal level.

At the herd level, after the first entry of the virus into the herd/holding through the infection of a few animals, and after the incubation period which had been previously reported for sheep pox (4–8 days) and for goat pox (5–14 days) [6]. In the case of the Spanish outbreak, it was estimated to be at about 14 days, based on the information gathered in the outbreaks, where only these few animals would show clinical signs, which is not at all alarming for the farmer, reducing the sensitivity of passive surveillance, especially in large herds with thousands of animals. These few animals would spread the virus throughout the holding, and after another incubation period of 14 days, there would be an explosion in clinical cases at least one month after the first entry of disease in the holding. We never observed an early explosion in the disease in any herd/holding, but a progressive and unobvious start, until the time when there was already many infected animals and high virus load in the environment, thereby increasing the chances for further silent spread to other farms, which occurred at least one month after the virus entered the farm. This fact may significantly jeopardize the early detection of the disease at the herd level and increase the possibility of spreading to other farms before the process is very evident in the affected herd.

On the other hand, at the animal level, only a small percentage of the clinically affected animals died from the infection. Although it is true that in most of the outbreaks, the disease was detected early, and the herd was immediately killed so the disease would not have time to evolve, in the few herds where the first detection was delayed, which was hinted at by the presence of many clinically affected animals during the first visit, the disease had time to evolve; however, explosive mortality of a large number of animals was not reported, but rather a low mortality rate, if any, affecting progressively a small number of animals in the herd, which was exposed to the same level of virus. Likewise, in no case was hyperacute mortality, without the development of clinical signs, reported.

**Figure 6:**
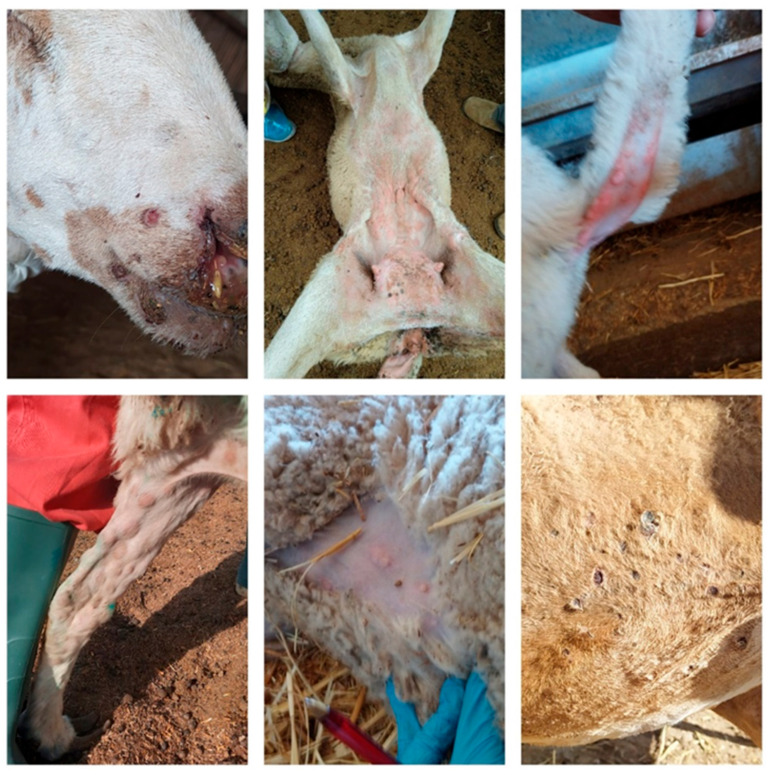
Skin signs and lesions characteristic of SGP in sheep in different stages of evolution, from early erythematous papules to necrotic nodules, to late dry rounded woodless scabs. (Source: OVS of AND and CLM).

Regarding the clinical signs at the animal level, the clinical presentation was fully compatible with what has been described in the WOAH manual and in the available scientific publications. Thus, the signs varied from mild to acute characterized by the appearance of fever, abatement, dyspnea/polypnea, conjunctivitis, tearing, rhinitis, eyelid oedema, photophobia, a rash that begins with erythematous rounded areas that turn in few days into papules, purulent ulcers due to bacterial secondary infection and, finally, dry scabs that would leave a wool-less round area when they had fallen from the animal. These typical skin lesions were particularly visible in the parts of the body without wool, such as the perineum, the inguinal area, the scrotum, the udder, the snout, the eyelids, and the armpits (Figure 6). In some cases, papules produced nodules that affected all layers of the skin and subcutaneous tissue, with secondary bacterial infections that worsened the lesions and the general condition of the animal. There was also inflammation and enlargement of the lymph nodes in some cases, accompanied by hemorrhagic congestion. Lung lesions also occurred, with severe and pox lesions, focal and uniformly distributed and visible throughout the surface of the lungs with congestion, oedema, focal areas of proliferation with necrosis and lobular atelectasis.

Therefore, from the point of view of passive surveillance, and in the frame of the awareness campaigns implemented to enhance it during the outbreak, it is important that all stakeholders in contact with the animals are aware of the characteristic clinical signs and lesions characteristic of the specific strain involved in the outbreak, and that any suspicions are immediately reported to the OVS, since passive surveillance plays a critical role in the early detection and efficient control of outbreaks. In this regard, it was important in the case of the Spanish outbreak, which could be different with other strains, to consider that the disease may not manifest itself clearly in the herd, particularly in large herds, until at least one month after the entry of the virus, and that it is therefore essential to have a thorough and frequent inspection of all animals, at least daily, to detect the disease as soon as possible. In the case of milk animals, milking is a good moment for this inspection, since the wool-less area under the tail is easily accessible and is an area where the lesions are usually present and very evident.

Finally, it should be noted that in the case of the outbreak in Spain, it was confirmed that it was a strain of sheep pox, which did not affect goats, even in mixed herds where goats lived with affected sheep. All samples of salivary/nasal swabs and whole blood from goats tested were negative by rPCR. However, following the requisites of Commission Delegated Regulation (EU) 2020/687, goats in confirmed mixed herds were killed with the sheep, and movement restrictions were applied on healthy goat holdings located in the adopted restriction zones.

### 3.2. Sampling Strategy in Restricted Zones and Samples of Choice for Confirmation of the Disease

Laboratory tests carried out in samples from the first confirmed outbreak identified that the specific strain present was a sheep pox strain. From these first moments, and following the recommendations of EU experts, it was established that although the virus was detectable in blood, the sample of choice in clinically affected animals was the swab of oral saliva or nasal exudate to carry out the rPCR since the virus would be detectable in saliva/nose exudate from a few days before the onset of clinical signs, and up to 80 days after infection. In the case of advanced lesions on the skin or scabs, the lesion smears would also have been an adequate sample.

In relation to the active surveillance strategy in the restricted areas, visits were carried out every 15 days to all holdings, under the appropriate biosecurity conditions, to inspect for the presence of clinical signs or compatible lesions in the animals and, in addition, bearing in mind that PCR could detect the infection a few days before the clinical signs, to systematically take samples of salivary swabs for PCR to increase the likelihood of early detection as much as possible, even if no clinical signs were detected in the animals. This system allowed for the very early detection of two outbreaks based on the positive results from animals that were not clinically affected but which showed clinical signs days later. However, at a later stage, the sampling of salivary swabs in official visits was only taken from those cases where compatible clinical signs or lesions were detected in the animals, to make for a more efficient use of resources and laboratorial capacity, especially in view of the extension in time and space of the restriction areas that had to be applied to ensure the effective control of the worsening situation. This later strategy was shown to be effective in controlling and finally eradicating the disease.

### 3.3. Awareness Campaigns among Farmers and Veterinarians Is Critical for Early Detection of New Outbreaks

Spain had been free from the disease since 1968. Due to the long period without epizootic outbreaks, we knew that, in the first moments, the level of awareness and knowledge regarding SGP would have been low in both livestock farmers and veterinarians.

Therefore, one of the main challenges in the management of the outbreak, especially at the beginning, was to increase the level of awareness about the clinical signs and the importance of the control of this disease in the shortest possible time, all with the aim of avoiding delays in the communication of suspicions of the disease which, in spite of the intensive awareness campaigns, were suspected to have occurred in some cases of the 7/2023 outbreak, which could have contributed to a longer time until the final control and eradication of the outbreak.

Awareness campaigns were carried out from the beginning and throughout the whole outbreak, focusing on farmers and veterinarians at both the central and regional levels.

Overall, the level of awareness of livestock farmers and veterinarians improved very soon after and was good during the outbreak in the whole country, particularly in the affected areas. This was monitored and evidenced by the high number of passively detected suspected cases throughout the country that were discarded as negative by PCR. In particular, during the outbreak period, 78 suspected cases (25 in AND, 38 in CLM, 2 in Castilla y León, 2 in Extremadura, 2 in Cataluña, 6 in Madrid, 1 in Navarra, and 2 in Valencia) were ruled out; in most cases, contagious ecthyma was confirmed as the cause of the observed clinical signs.

In some cases and zones, delayed detection occurred due to the following: (1) most of the cases occurred in holdings with very high census (several thousand animals) where it was difficult to detect clinical signs, and the lesions were only apparent in very few animals in those initial moments; this is suspected to have occurred in the 2023/1 and 2023/3 outbreaks; (2) In a few cases, the OVS had found evidence of intentionally delayed communication by a few operators, as in the 2023/7 outbreak.

### 3.4. Farm and Animal Transport Biosecurity Is Fundamental for the Control of the Outbreak

The SGP virus is highly resistant in the environment, particularly when scabs develop in the affected animals and fall on the ground, where the virus can remain viable for months, thus biosecurity at all levels is a key factor for the effective control of this disease.

Biosecurity was identified, based on the epidemiological investigations carried out during the outbreaks, as one of the most important factors that could be a contributing factor to the spread of the disease in the affected areas. The most important biosecurity aspects identified included the following: (1) lack of fences, fences in inadequate condition, or failure to comply with appropriate cleaning and disinfection protocols between dirty and clean areas delimitated by the fences; (2) entry of external vehicles into the holdings without adequate cleaning and disinfection conditions, mainly lamb collection trucks for the transfer of lambs to the slaughterhouses, which was identified as the most likely route of transmission in many outbreaks, mainly in CLM, or in a few cases, the milk collection truck for the transfer of milk to the processing plant, as well as the feed delivery truck; (3) handling of animals without the application of the appropriate biosecurity protocols by a truck driver from outside the holding who had finally selected the lambs to be transported to the slaughterhouse, although in some cases, this was the possible role of the shearers, even though shearing was not linked to any outbreak in the epidemiological investigations that were carried out on them; and (4) direct or indirect contact between herds in the common pastures and paths used by the different herds, particularly in herds which were without pastures for their individual use that had to move to common pastures; this situation was especially common in the affected area of Andalucía before the application of the restricted zones. All these factors could contribute to the risk of spreading the virus to healthy farms thus producing secondary outbreaks.

In relation to factors 2 and 3 which, again based on the findings brought about by the epidemiological investigations carried out, were the most likely routes of transmission between most of the affected holdings, especially in the affected areas in CLM where there were a high number of epidemiological links by this route, it is important to note that a common practice in the sheep and goat sector would have been the movement of the same lorry between several holdings to collect the lambs until the lorry was completely loaded, only then would the final transfer to the slaughterhouse be carried out for the slaughter to take place. Sometimes, these trucks would access the production units of the farm without adequate biosecurity protocols in place, and the truck drivers may have then entered the production units to select the lambs to be transported. Thus, it could happen that the truck would access an infected holding, in which the virus had not yet been detected, to carry out the collection of animals, after which it would travel to other healthy farms without the application of appropriate biosecurity protocols, resulting in a silent spread of the virus.

To try to control the risks associated with these factors, several measures were put in place and contributed greatly to the effectiveness of the control of the disease in the affected areas, among which we highlight the following:

- Development of specific protocols for risky activities such as the collection of lambs, collection of milk, shearing, etc.
- Meetings with the sector and organization of awareness-raising campaigns for farmers and private veterinarians in the affected areas and throughout the country, to raise awareness of the importance of strict biosecurity measures both on farms and in animal transport.
- Implementation of specific official controls to ensure the adequate cleaning and disinfection of each vehicle arriving at the slaughterhouse with animals from the restricted zone. This was complemented by a control carried out on the animals regarding the number and traceability, and with additional controls implemented on every single load of lambs in the different farms of origine. This measure was very resource-demanding but was shown to be very effective in improving the bad epidemiological situation in CLM.
- This risk of outdoor common pastures was considered in the epidemiological investigations carried out during the outbreaks; therefore, the awareness of the farmers in Andalucía was raised to maintain their animals indoors and not take them to the common pastures, and as this was contrary to the traditional grazing practices in these areas, the OVS decided to support the farmers in the feeding of their animals indoors, which contributed greatly to the compliance with this measure.

### 3.5. Strict Control on Movement of Animals in Problematic Affected Areas Played a Critical Role to Control the Outbreak

Another challenge, particularly in the affected area of CLM, as this area had a high level of commercial activity, was the control of the health documents required by the relevant authorities for the movement of animals within the restricted areas, as well as between this area and the free areas. Thanks to these controls, few cases were detected of the movement of lambs and sheep without compliance with these provisions. In these cases, the farmers involved were subject to prosecution by the OVS. These controls were key to keeping the spread of the disease under control in the affected areas.

To ensure that these controls were effective, investigations conducted at the local level and the identification of the operators suspect of breaking rules in the affected areas were key, as well as the day-to-day communication and coordination with the security forces, particularly the Traffic Guard and the SEPRONA, a section of the Civil Guard dedicated to crimes against the environment. The OVS reported, whenever necessary, the suspected operators and registration plates of vehicles to the security forces who then implemented targeted controls in the area, focusing on these operators and vehicles.

### 3.6. Progressively Increasing Size and Duration of Restricted Zones to Adapt to the Worsening Situation Was Very Effective in the Control of the Outbreak in Problematic Affected Areas

To ensure the control of the spread of the virus, especially after the detection of cases which happened with abnormal disease jumps in space (outside the previously established RZ) and time (later than the maximum incubation period of the disease after the last outbreak detected in the area) and possibly due to human factors, a progressive increase in the size and duration of the restriction zones was implemented.

**Figure 7:**
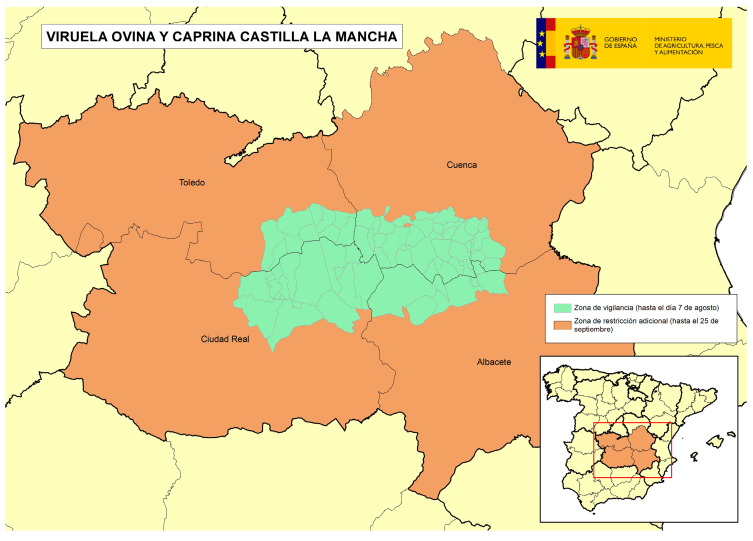
Last restriction zone adopted in CLM grouping the outbreaks detected in Alcázar de San Juan (Ciudad Real, outbreaks 2023/3–6) and Casas de Fernando Alonso (Cuenca, outbreak 2023/7) (source: OVS CLM and MAPA).

Initially, a PZ with a radius of 3 km was adopted for a total of 21 days, as well as an SZ with a radius of 10 km for a total of 30 days, in accordance with European Union legislation. However, given the unfavorable evolution of the epidemiological situation, it was decided to have both these areas extended in size and duration, and this happened in progressive and successive extensions until reaching a final RZ that included a PZ composed of all municipalities within a 10 km radius around the outbreak for a total of 48 days, and an SZ composed of all municipalities within a 30 km radius around the outbreak for a total of 66 days (Figure 7). In these areas, the restriction and the controls were also progressively enhanced to minimize the risk of spreading the virus outside the RZ.

**Figure 8:**
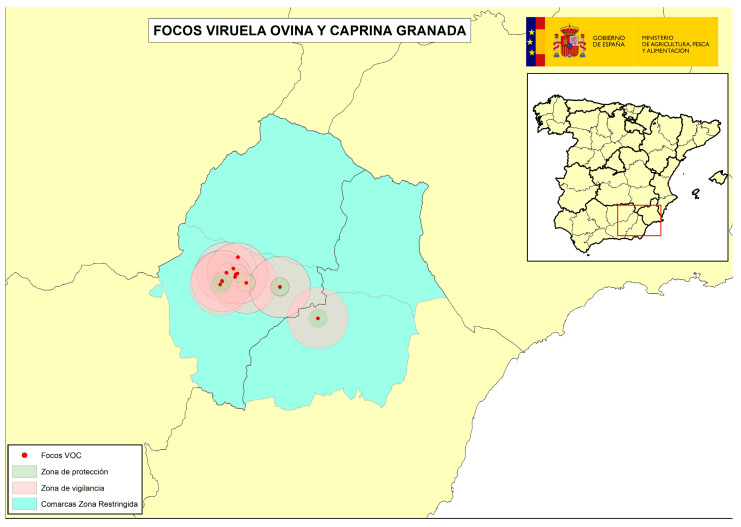
Further RZ adopted in Andalucía including 4 districts (source: OVS AND and MAPA).

**Figure 9:**
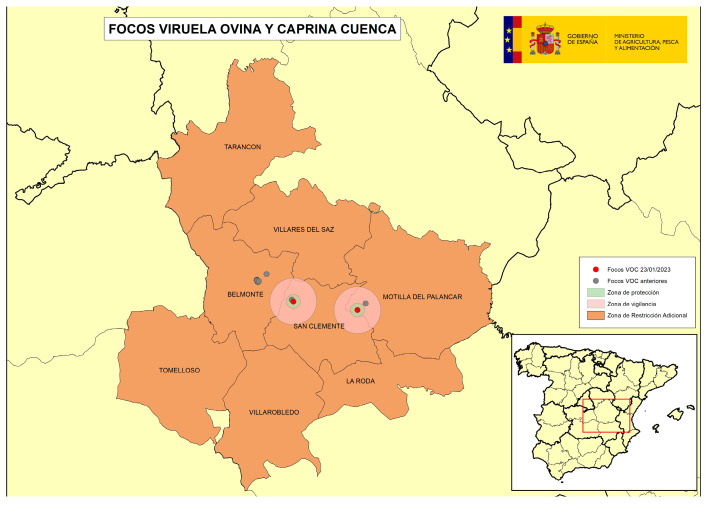
Initial Further RZ adopted in CLM including 8 districts (source: OVS CLM and MAPA).

**Figure 10:**
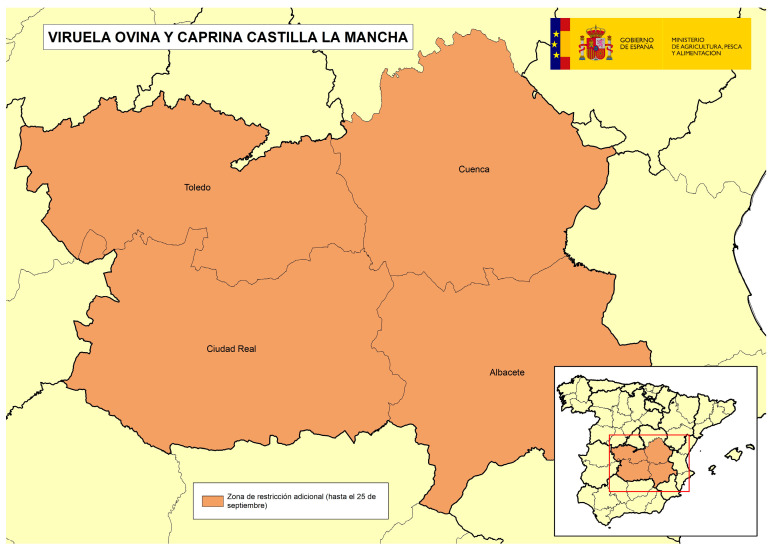
Final Further RZ adopted in CLM including four full provinces (source: OVS CLM and MAPA).

In addition to the RZ, it was decided to adopt a Further RZ in both the affected Autonomous Communities, to increase health guarantees in the commercial movements of live animals from this Further RZ to the free zone, helping to ensure that the virus did not escape the affected areas. In the case of AND, this Further RZ included four districts (Figure 8), and in the case of CLM, there was an initial Further RZ including eight districts where epidemiological links between farms existed or were suspected (Figure 9), after which, due to the unfavorable epidemiological evolution, the zone was extended to include four full provinces (Cuenca, Albacete, Ciudad Real and Toledo) (Figure 10).

The implementation of this enhanced regionalization clearly contributed to the effective control of the outbreak.

## 4. Conclusions

Throughout the outbreak, a number of factors/aspects were detected that were contributing factors to the ineffectiveness of the control measures put in place. A constant analysis of the situation and the epidemiological information from the affected holdings and areas enabled the detection of these factors/aspects. For the control of these factors/aspects, corrective actions were implemented that contributed to the effective control of the outbreak and final eradication of the disease in Spain.

The most relevant factors/aspects, given the challenges in the management of the outbreak, and on which the corrective actions were implemented which contributed to the effective control of the outbreak in Spain thus representing the lessons learnt for the management of future outbreaks, included the following: Firstly, the adoption, through awareness campaigns, of passive surveillance to the presentation of the disease at a herd level that was unclear and difficult to detect early. For this purpose, farmers and veterinarians were urged to closely and regularly monitor the animals to detect any signs of the disease as soon as possible. Second, active surveillance in restricted areas was adapted to the evolving disease situation, assessing the cost and benefits of the strategies implemented and trying to ensure the most efficient use of the resources available for control, particularly the diagnostic capacity of the Algete NRL. Thirdly, a key aspect was to increase the level of biosecurity awareness which, in many sheep farms, was not very high; this was a key aspect in the control of the disease. Fourthly, close collaboration and coordination between the OVS and local security forces, mainly SEPRONA and the Traffic Guard, was key to controlling certain operators, especially in CLM, who were performing illegal activities and who were causing problems for the final control of the outbreak. Finally, the adaptation, throughout the outbreak, of the restricted zones applied to the disease situation, which were increased in size and duration to try and place the situation under control due to the worsening of the situation, especially in CLM where the outbreak lasted longer. Through the application of the different measures/corrective actions described throughout this text, we could finally achieve complete control and eradication of the disease in Spain.

In conclusion, Spain’s experience in managing this outbreak involved the detection of factors/aspects that were undermining the effectiveness of the control measures implemented. Its detection allowed for the identification and implementation of corrective actions/measures to achieve effective control of the disease in the shortest possible time.

This article therefore is intended to describe the experience of Spain in the management and control of the outbreak of SGP, with the description of the situation, the identification of the main problems, challenges and difficulties encountered, as well as the different solutions or corrective actions/measures implemented to solve them, in order to achieve effective control of the disease in the shortest possible time. The problems identified and the corrective measures put in place to address them are valuable lessons learnt for the effective and more efficient control of future outbreaks.

## Figures and Tables

**Table 1:** Outbreak ID, location, animal census, number (Nº) of animals clinically affected and dead at first official visit and date of notification of the total 30 SGP outbreaks confirmed in Spain in 2022–2023 (source: OVS AND/CLM, and MAPA).

Outbreak ID	Autonomous Community	Province	Municipality	Animal Census (Sheep/Goat)	Nº Animals Clinically Affected at First Official Visit	Nº Animals Informed Dead in the epi Investigation at First Official Visit	Date of Notification
2022/1	Andalusia	Granada	Benamaurel	314/11	50	30	19 September 2022
2022/2	Andalusia	Granada	Cúllar	170/20	1	0	26 September 2022
2022/3	Castilla–La Mancha	Cuenca	Villaescusa de Haro	890/0	1	0	26 September 2022
2022/4	Castilla–La Mancha	Cuenca	Villaescusa de Haro	7654/0	182	0	27 September 2022
2022/5	Andalusia	Granada	Benamaurel	340/14	2	0	29 September 2022
2022/6	Castilla–La Mancha	Cuenca	Villaescusa de Haro	227/0	1	0	29 September 2022
2022/7	Castilla–La Mancha	Cuenca	Villaescusa de Haro	1877/0	8	0	29 September 2022
2022/8	Castilla–La Mancha	Cuenca	Villaescusa de Haro	5075/0	15	0	29 September 2022
2022/9	Castilla–La Mancha	Cuenca	Villaescusa de Haro	591/0	15	0	29 September 2022
2022/10	Andalusia	Granada	Benamaurel	110/6	3	0	4 October 2022
2022/11	Andalusia	Granada	Benamaurel	79/6	1	0	4 October 2022
2022/12	Andalusia	Granada	Benamaurel	639/20	7	0	6 October 2022
2022/13	Andalusia	Granada	Cúllar	192/9	50	0	11 October 2022
2022/14	Andalusia	Granada	Cúllar	42/0	4	0	11 October 2022
2022/15	Castilla–La Mancha	Cuenca	Villaescusa de Haro	7354/0	2	0	13 October 2022
2022/16	Castilla–La Mancha	Cuenca	Villaescusa de Haro	3591/0	10	0	13 October 2022
2022/17	Andalusia	Granada	Baza	373/0	1	0	19 October 2022
2022/18	Andalusia	Almería	Oria	97/9	15	0	2 November 2022
2022/19	Andalusia	Granada	Benamaurel	364/30	30	0	2 November 2022
2022/20	Andalusia	Granada	Benamaurel	206/26	20	0	8 November 2022
2022/21	Andalusia	Granada	Benamaurel	149/18	1	0	8 November 2022
2022/22	Castilla–La Mancha	Cuenca	Alberca de Záncara	1519/0	15	5	29 November 2022
2022/23	Castilla–La Mancha	Cuenca	Tébar	820/0	30	15	29 November 2022
2023/1	Castilla–La Mancha	Cuenca	Alberca de Záncara	1359/311	50	0	23 January 2023
2023/2	Castilla–La Mancha	Cuenca	Tébar	3544/0	1	0	23 January 2023
2023/3	Castilla–La Mancha	Ciudad Real	Alcázar de San Juan	8100/0	480	5	8 February 2023
2023/4	Castilla–La Mancha	Ciudad Real	Alcázar de San Juan	1216/0	4	0	22 March 2023
2023/5	Castilla–La Mancha	Ciudad Real	Alcázar de San Juan	1410/0	5	0	30 March 2023
2023/6	Castilla–La Mancha	Ciudad Real	Alcázar de San Juan	3142/260	1	0	10 April 2023
2023/7	Castilla–La Mancha	Cuenca	Casas de Fernando Alonso	334/27	21	2	17 May 2023
TOTAL	—	—	—	51,778/767	1026	57	
